# Capsular Weakness around Breast Implant: A Non-Recognized Complication

**Published:** 2015-07

**Authors:** Pedro Salinero Arquero, Fabiana Cristina Zanata, Lydia Masako Ferreira, Fabio Xerfan Nahas

**Affiliations:** 1 Clinica Dr. Pedro Arquero, Madrid, Spain; 2 Division of Plastic Surgery, Federal University of São Paulo, São Paulo, Brazil

**Keywords:** Capsular weakness, Dynamic deformity, Capsulorrhaphy, Capsuloplasty, Breast augmentation

## Abstract

Capsular contraction is a frequent complication following breast augmentation. On the other hand, capsular weakness, a not widely recognized complication, may occur around the implant. A weak capsule allows the migration of the prosthesis to the lateral region of the thoracic region or inferiorly, towards the abdomen, due to gravitational forces. The cause of capsular weakness remains unresolved. Implant malposition, with lateral or downward displacement, breast asymmetry, improper contour, with implants moving in the pocket that compromise the aesthetic outcome of breast augmentation and require surgical correction may be different symptoms from the same clinical problem. Capsular weakness is a short or mid-term complication of breast augmentation. Most techniques aim to correct the malposition by making sutures to increase the resistance to the displacement of the implant, rearrange the structures using the capsule as flaps to remodel the envelope of the new pocket, obtaining a more stable and reliable result. In this article, four cases of displacement of breast prosthesis with capsular weakness are described and the surgical treatment that included a capsulotomy and capsulorraphy is described.

## INTRODUCTION

Capsular contraction is a frequent complication following breast augmentation and it was well described by Baker including the clinical aspects of hardness, stiffness and thickness of the capsule.^1^^-^^3^ As new technologies emerged in the 1990s, such as the development of textured implant shells and the use of cohesive silicone gel, there was a decreased incidence of this complication. In the year 2000, the Institute of Medicine (IOM) released its report on complications resulting from the placement of silicone gel breast prostheses. The committee’s work resulted in a 440-page report covering all aspects of silicone breast implants publications prior to that year, finding a capsular contracture rate ranging from 8% to 41%.^4^ The Mentor and Allergan pre-market approval studies, with 1007 and 940 women respectively, described the capsular contracture rate for saline and silicone gel implants as 15% on a strict follow-up of 10 years.^5^^-^^9^

On the other hand, the Baker classification did not mention the other side of the problem, a not widely recognized complication, the capsular weakness. Implant malposition, lateral or downward displacement, breast asymmetry, improper contour were consequences of capsular weakness that compromise the aesthetic outcome of breast augmentation and usually require surgical correction.^2^^,^^10^^,^^11^ The weak capsule allows the migration of the prosthesis to the lateral region of the thoracic region or inferiorly towards the abdomen due to gravitational forces. In such cases, the prostheses will partially occupy an abdominal position, the areola will be facing upwards and the patient would present an increased distance between the nipple and the infra-mammary fold, making the appearance of bottoming down or double bubble.^12^

As an intra-operative finding, macroscopically the surgeon can observe, in cases of capsular weakness, that the capsule is thin and lax. In some cases, it is possible to reach the glandular tissue through the capsule by blunt dissection. No matter numerous theories, the cause of capsular weakness remains unresolved. However, it is important to recognize and treat this condition.

## CASE REPORT

*CASE 1*

**Fig. 1:**
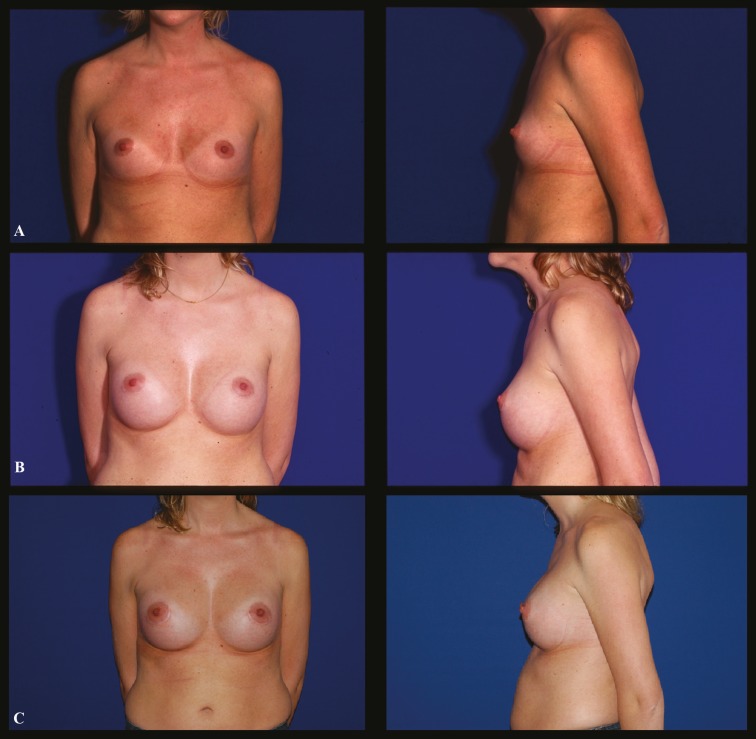
Case 1 (Frontal and profile view): **A.** Preoperative aspect of a 25 year-old female who presented hypotrophyc breasts. **B.** Six-month postoperative views after the first breast augmentation with a 400 ml round smooth silicone filled implants, moderate profile, presenting capsular weakness and lowering of both infra-mammary folds. **C.** Sixteen-month postoperative views after a capsulotomy and capsulorraphy with reinsertion of the implants

A 25-year-old white female who presented breast hypotrophy was undergone breast augmentation with 400 ml round smooth, moderate profile silicone implants inserted through an inferior periareolar incision. Implants were placed in a retropectoral pocket. After 6 months, she presented for consultation complaining about bottoming down of both implants. A revision surgery of both breasts was performed trough an infra-mammary incision. A capsulectomy of the lower pole associated with a capsuloplasty and capsulorraphy in the area of the inferior pole was done, defining the new inframammary fold. Breast implants were not changed (Figure 1).

*CASE 2*

**Fig. 2:**
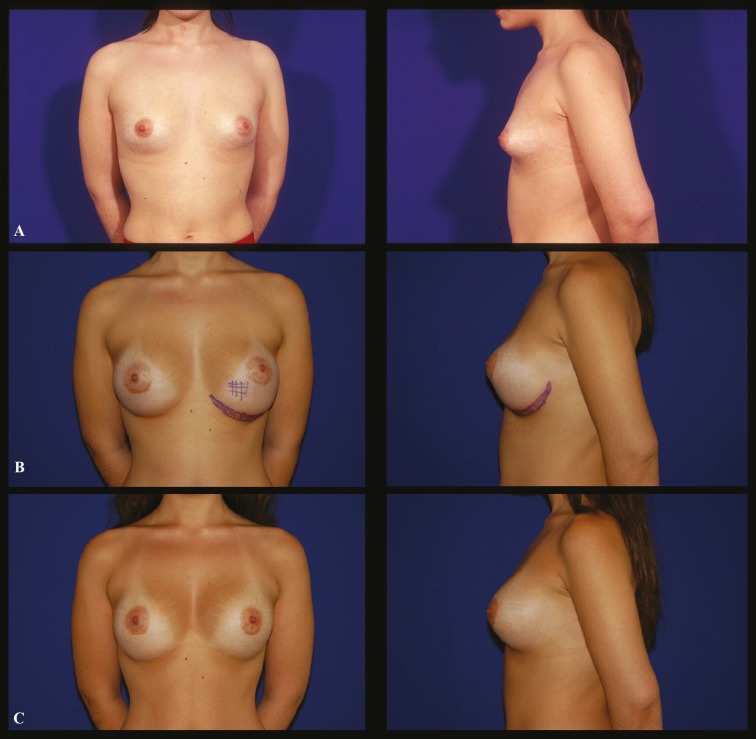
Case 2 (Frontal and profile view): **A.** Preoperative aspect of a 19 year-old white female who presented a left tuberous breast. **B.** Seven-month postoperative aspect after the insertion of a 325 ml round smooth silicone filled implants, showing an evident displacement of the left prosthesis to the abdominal region. **C.** Nine-month postoperative aspect after a capsuloplasty of the left breast

A 19 year-old white female who presented breast hypotrophy with left tuberous breast was undergone breast augmentation with a 325 ml, round smooth, high profile silicone implants, through an inferior periareolar incision. Implants were placed in a retropectoral pocket. One month after surgery, she complained about bottoming down of the left implant. A capsulectomy with removal of the area superiorly to the new infra-mammary fold was performed on the left breast and a capsulorraphy was made at the left breast to define the new inframammary fold (Figure 2).

*CASE 3*

**Fig. 3:**
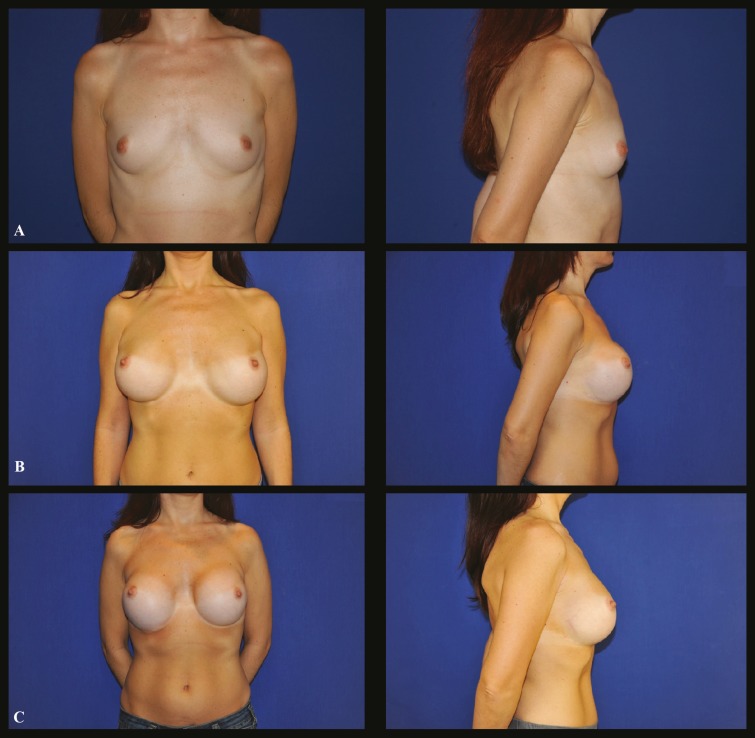
Case 3 (Frontal and profile view): **A.** Preoperative aspect of a 31 year-old white female who presented hypotrophyc breasts. **B.** Four months after the surgery with displacement of the implants. **C.** Twelve months postoperative aspect after revision of both breasts with capsulotomy and capsulorraphy to redefine the inframammary folds. Note the new position of both inframammary folds

This 31 year-old white female came for consultation after 4 surgeries performed somewhere else. She had a 450 ml, round textured implants ultra-high profile silicone implants, inserted, first through axillary incisions and after trough infra-mammary incisions. Implants were placed on a retropectoral pocket. Four months after the surgery, she complained about bottoming down of the right implant. A capsulotomy and capsulorraphy were performed to define the new inframammary fold (Figure 3).

*CASE 4*

**Fig. 4:**
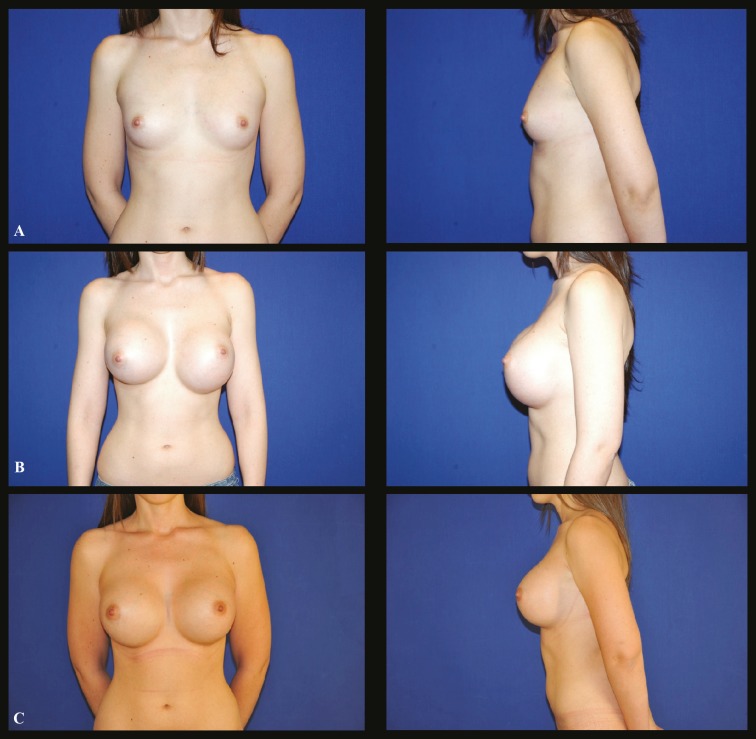
Case 4 (Frontal and profile view): **A.** Preoperative aspect of a 25 year-old white female who presented breast hypotrophy. **B.** Six months postoperative aspect after a 400 ml round smooth silicone filled implants, showing an evident displacement of the left prosthesis to the abdominal region. **C.** Eighteen months postoperative aspect after a capsulotomy at the right breast and capsuloplasty at the left breast

A 25 year-old white female who presented breast hypotrophy was undergone breast augmentation with a 400 ml round smooth, ultra high profile silicone implants inserted through infra-mammary incisions. Implants were placed in a retropectoral pocket. After 6 months, she presented for consultation complaining about bottoming down of the left implant and capsular contraction in the right implant. A revision surgery of both breasts was performed with capsulotomy on the right breast and capsulotomy with capsulorraphy on the letf, both in the area of the inferior pole, along the new infra-mammary fold. Breast implants were not changed (Figure 4).

## DISCUSSION

Revision in breast augmentation surgery is a persistent and recurrent challenge to plastic surgeons. It is a complex procedure in secondary surgeries, with unpredictable results, especially in demanding patients.^10^^,^^11^^,^^13^ Although capsular contracture is a very well described complication with an objective classification, capsular weakness can have different clinical aspects and surgical approaches.^3^

The senior surgeon often noted that capsular weakness is more frequently observed when smooth implants were used regardless its location (subglandular or retropectoral). The capsule is well formed, with a sinovial aspect, with or without some liquid in the pocket. It is not different from a normal and flexible breast, classified as Baker I.^3^ Furthermore, this condition is probably due to the mobile condition of these implants as opposed to the Velcro effect that occurs when textured or poliurethane implants were used. Prosthesis mobility tended to expand the pocket in lax capsules. 

Histological studies evaluated the amount of collagen type I and III and very large differences were found between different capsules.^14^^-^^17^ Type I collagen is a more structural and strong collagen whereas type III is a more flexible fiber.^18^ This must be interpreted as differences among the stiffness or compliance of the capsule.^19^ Therefore, not all capsules are the same. Further studies in clinical cases must be done to evaluate the differences between capsular contracture and capsular weakness. 

The prosthesis displacement may occur due to gravity or muscle activity associated with a lax capsule. It can be applied a basic law of the mechanics of materials in the breast. As the breast, is a viscoelastic material, when the breast is loaded with an implant, it produces a stress that causes the breast to deform obtaining the augmentation. This behavior might be graphed in a theoretical material’s stress-strain curve. This deformation will increase with time although the implant remains constant. This is a concept termed “creep deformation” on material science.^19^

If the capsular contraction does not happen, this stress continues to deform the pocket enlarging the collagen fiber to the point of the elastic limit or yield point. The cells multiplication and the production of new fibers enlarge the existent tissue, making a permanent deformity, thus increasing the size and extension of the pocket. The weakened tissue allows the implant to move and increases the pouch.^14^^,^^15^^,^^17^

The patients reported in this paper correspond to the type 2 by Massiha’s classification of the Double Bubble deformity,^12^ explained as high-compliance, low-stiffness/low-resilience breasts, with empty hypoplastic breast after pregnancy or nursing (most of the augmentation-mastopexy procedures could be in this category). With lack of tissue support, this type of breast can cope with a large and high profile implants. Because of its low resilience, it might suffer a considerable amount of creep deformation on a long-term follow up.^19^^,^^20^

It is impossible to predict which patients will develop capsular contracture. Similarly, it is not possible to evaluate which patients will present capsular weakness. The correction of both complications needs a second operation. The correction of this type of deformity can be achieved by using sutures attaching the subcutaneous tissue and capsule to the pectoralis fascia, thus recreating the sub mammary sulcus. In cases of lateral positioning of the prosthesis, sutures can limit the lateral aspect of the pocket.^11^^,^^20^^-^^23^

The use of the capsule on secondary cases should be considered. Although in these cases there is a capsular weakness, a mature capsule is more likely to have a stable fibrosis and could hold the implant in place. Capsular weakness, a short or midterm complication of breast augmentation, should be identified. To use sutures to limit the displacement of the implant and to reduce pocket size by using the mature capsule as flaps to correct prosthesis malposition are efficient techniques to deal with this complication. 

## CONFLICT OF INTEREST

The authors declare no conflict of interest.

## Note

Please cite this paper as:

Arquero PS, Zanata FC, Ferreira LM, Nahas FX. Capsular Weakness around Breast Implant: A Non-Recognized Complication. World J Plast Surg 2015;4(2):168-174.
